# Improving Integration of Palliative Care and Nephrology

**DOI:** 10.34067/KID.0000000666

**Published:** 2025-02-27

**Authors:** Amar D. Bansal

**Affiliations:** Renal Electrolyte Division, Section of Palliative Care and Medical Ethics, University of Pittsburgh School of Medicine, Pittsburgh, Pennsylvania

**Keywords:** CKD, geriatric nephrology, patient-centered care, conservative management, supportive care

CKD is a serious illness with high symptom burden, difficult treatment decisions, and low rates of advance care planning (ACP) that contribute to intense end-of-life care. These factors are particularly relevant because those with CKD tend to be older persons with high rates of multimorbidity. Improving integration of palliative care (PC) and kidney care is therefore an important way to fulfill unmet needs.

In oncology, there has been substantial growth in early PC for patients with metastatic cancer. Studies in that setting have shown that early PC involvement can lead to improvements in quality of life, mental health symptoms, and even increased survival when PC is provided alongside oncology care.^1,2^

To what extent analogous efforts could lead to improvements in outcomes in patients with CKD remains an area of active exploration. A variety of PC interventions have been implemented and described in the outpatient setting, including noteworthy innovation in the overlap between dialysis care and hospice.^3–5^

To further explore whether integration of PC into kidney care would be feasible and acceptable, Saeed *et al.* conducted a pilot randomized control trial involving patients 75 years and older with advanced CKD reported in this issue of *Kidney360*.^6^ Their intervention was termed Enhanced Dialysis Education for CKD (CKD-EDU) and focused on patients with eGFR ≤25 ml/min. Patients randomized to the control arm continued usual care with their nephrologist. Patients in the intervention group received educational booklets about kidney disease treatment options along with a list of questions related to treatment options and ACP topics. The intervention patients met with the study research coordinator who recorded their hopes, goals, and fears about kidney treatment options and provided the form to PC interventionists. Patients met with a PC clinician for up to three visits, each approximately 4–6 weeks apart. Those visits were semistructured and designed to continue discussions related to treatment decisions and ACP. The main outcomes were whether the CKD-EDU intervention would be feasible and acceptable. Feasibility was defined as success in patient recruitment, randomization, and overall reliability of follow-up measurements. Acceptability was measured using a specific tool named Acceptability of the Intervention Measure. Comparisons between how standard care versus CKD-EDU affected decisional conflict and quality of life were also made.

Of the 58 patients who consented to randomization, 30 were allocated to the CKD-EDU arm. The authors reported an enrollment rate of 44% of potentially eligible patients and over 90% retention from assessments made at later time points in the study. These findings led them to conclude their study was feasible. Acceptability measures were high with patients in CKD-EDU reporting 86% likelihood or high likelihood that they would recommend the PC services to others.

Among the secondary outcomes examined, they reported improved results on the decisional conflict scale at the 4–6-week assessment between the intervention and control groups. This finding was true for both patients and caregivers. In addition, scores for the Burden of Kidney Disease Subscale were also significantly lower in the CKD-EDU group at the 24–26-week assessment when compared with the control group. A variety of other comparisons all showed desirable, but nonsignificant, trends in the intervention group.

The findings from this work have important implications. Whether the PC intervention would be rated as acceptable by patients was one of their primary outcome measures, and the positive result is itself noteworthy. In addition, the CKD-EDU intervention provided patients and families with additional information about their treatment options. This information and the ensuing semistructured discussions with PC clinicians likely helped participants establish a kidney therapy decision—presumably choosing between dialysis versus conservative kidney management (CKM). Although not explicitly mentioned, skilled conversations with PC clinicians in the intervention arm likely resulted in improved *coping* for patients and caregivers as they navigated uncertain trajectories.^7^

Owing to the understandably limited scope of this pilot randomized control trial as an assessment of feasibility and acceptability, it leaves many important questions unanswered that will hopefully become clearer in future work. First, to what extent health systems are willing to invest additional resources to bring PC to high-risk patients with kidney disease is unknown. The answer likely varies at the individual system level, but considering this uncertainty will be an important first step in planning for real-world implementation and scalability. Interventions like the ones proposed in this study require an acknowledgment that real trade-offs exist when additional health care resources are used. Potential benefits that arise because of PC interventions may not be easy to capture. Specifically, health systems will be likely interested in gauging whether investing additional resources could lead to the delivery of more high-value care, such as reducing hospitalization rate or shortening hospital length of stay. How these metrics are prioritized will vary, but there will probably be an interest in determining to what extent integration of PC into kidney care might reduce dialysis initiation rates for patients whose values align with CKM. Recent data and accompanying publicity questioning the benefit of dialysis for older adults might bring this question to the forefront.^8,9^

Another area that will need attention is how to precisely define the target population that could benefit from a PC-based intervention. In this study, 40 patients were excluded from the study because they already met with a dialysis educator. This number represents a sizable proportion of the entire eligible study cohort and could reflect a bias among nephrologists who already (paternalistically) decided that dialysis is the appropriate therapy for their patient. In other words, dialysis had been framed as the default choice, or the only choice for these patients. Their ensuing interaction with the dialysis educator therefore resulted in them being excluded from the study. Thinking of ways to prevent so many patients from being bypassed would be a worthwhile undertaking for a future study. Because many clinicians view dialysis initiation as a sign of success, increasing the visibility of CKM as a viable treatment option will therefore need to be one of the essential consequences of better integration between nephrology and PC.^10^

Furthermore, the design of future study cohorts will need to incorporate the slow progression of CKD in older patients. If outcomes such as progression to dialysis initiation, opting for CKM, or death would potentially be measured, then the event rate will need to be high enough for meaningful conclusions to be obtained. For example, could the addition of impaired functional status as an inclusion criterion be a useful way to identify patients at highest risk of faster overall decline? Doing so could also have the added benefit of reducing the likelihood that nephrologists would exclude patients from study interventions because they would represent a frailer subset.

Finally, because many older patients with CKD initiate dialysis, how future studies might navigate this transition will be an area of interest. Will the medical siloing and bureaucratic changes that accompany this transition denote the end of the PC intervention? This conundrum highlights the challenge of integrating PC into two distinct care models within nephrology: predialysis kidney care in an outpatient clinic and dialysis care for patients on treatment with chronic dialysis.

With these considerations in mind, it will be exciting to see how PC interventions might alleviate some of the burdens shouldered by older adults living with CKD and their caregivers. Integration of PC into nephrology will require time, patience, trust, and reimagining what success looks like. Nevertheless, this effort holds the promise of a more responsive and person-centered approach to kidney care, aligned closely with the unique needs and priorities of older patients. Over time, integration could also augment *primary* PC skills within the nephrology workforce, thereby leading to a more patient-centered approach than what is currently offered.
